# Risk factors for distant metastasis in locoregionally controlled oral squamous cell carcinoma: a retrospective study

**DOI:** 10.1038/s41598-021-84704-w

**Published:** 2021-03-04

**Authors:** Hirofumi Tomioka, Yuko Yamagata, Yu Oikawa, Toshimitsu Ohsako, Takuma Kugimoto, Takeshi Kuroshima, Hideaki Hirai, Hiroaki Shimamoto, Hiroyuki Harada

**Affiliations:** Division of Oral Health Sciences, Department of Oral and Maxillofacial Surgery, Medical and Dental Sciences, Graduate School of Medical and Dental Sciences, Tokyo Medical and Dental University, 1-5-45 Yushima, Bunkyo-ku, Tokyo, 113-8549 Japan

**Keywords:** Surgical oncology, Oral cancer

## Abstract

The control of distant metastasis in oral squamous cell carcinoma is an important determinant of improved prognosis. The study aimed to identify risk factors for distant metastasis in patients with locoregionally controlled oral carcinoma. We identified 982 patients with oral squamous cell carcinoma treated at our hospital between January 2008 and December 2017. After excluding patients with distant metastasis at initial treatment, patients with metastasis to the oral cavity, those receiving palliative treatment, and those lacking follow-up data, 941 patients were selected. Finally, among these 941 patients, 887 with locoregionally controlled oral squamous cell carcinoma were included in the study. Among the 887 patients, 36 had confirmed distant metastasis (4.1%), and the lung was the most common site (31/36 patients, 86.1%). Multivariate analysis showed that the incidence of primary intraosseous carcinoma of the mandible, cervical lymph node metastasis at levels IV and V, and the presence of pathological extranodal extension were significant risk factors for distant metastasis. When treating patients with oral squamous cell carcinoma who are positive for the aforementioned risk factors, the possibility of developing distant metastases must be accounted for, and aggressive treatment should be planned accordingly.

## Introduction

The control rate for primary and cervical lymph node metastases has been gradually improving in patients with oral squamous cell carcinoma (OSCC) owing to improved treatments. However, the incidence of distant metastases has been increasing, and the occurrence of distant metastasis in OSCC is often associated with reduced survival^1^. Therefore, it is essential to identify such high-risk patients with distant metastasis in advance to provide effective treatment as early as possible. The incidence of uncontrolled primary tumor and cervical lymph node metastases may lead to distant metastases during the development of these recurrent tumors. Investigation of these cases will not be beneficial in predicting or preventing distant metastasis. Thus, in this study, we aimed to identify and examine the high-risk factors for distant metastasis by including only patients with OSCC who achieved locoregional control.

## Results

### Patient characteristics

**Figure 1:**
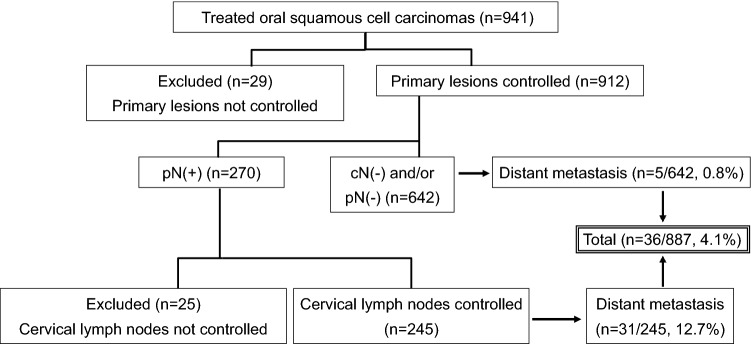
Flowchart depicting the patient selection process.

Among 941 patients with OSCC, 29 in whom the primary tumor recurred after the initial treatment and could not be relieved by subsequent surgery or radiotherapy, resulting in uncontrolled primary tumor, were excluded from the study. Among 912 patients in whom the primary tumor was controlled, 270 (29.6%) had histological cervical lymph node metastases and 642 had clinically and/or histologically negative lymph nodes (Fig. 1). In addition, among 270 patients with cervical lymph node metastases, 25 with uncontrolled cervical lymph node metastases were excluded. Among 245 patients with cervical lymph node metastasis, distant metastasis was confirmed in 31 (12.7%) patients; on the other hand, among 642 patients without cervical lymph node metastasis, distant metastasis occurred in only 5 patients (0.8%).

Finally, among the 887 patients with locoregional control who were examined, 36 (4.1%) developed distant metastases. The 36 (27 male and 9 female) patients were aged 24–87 years with a mean age of 64.4 years. According to the primary tumor site, the rate of distant metastasis was the highest in primary intraosseous carcinoma (PIOC) of the mandible (4/16 patients, 25.0%), followed by buccal mucosa (6/90 patients, 6.7%) and tongue (19/516 patients, 3.7%) carcinomas (Table 1).

**Table 1:** Incidence of distant metastases based on primary site.

Primary site	Incidence of distant metastasis	%
Tongue	19/516	3.7
Floor of the mouth	1/45	2.2
Upper gingiva	3/87	3.4
Lower gingiva	3/124	2.4
PIOC of the mandible	4/16	25.0
Buccal mucosa	6/90	6.7
Hard palate	0/9	0.0
Total	36/887	4.1

PIOC, primary intraosseous carcinoma.

The clinicopathological features and results of the univariate analysis are shown in Table 2. The initial treatment for the primary tumor was surgery in 775 patients and brachytherapy (Cs-137, Ir-192, or Au-198) in 112 patients. Brachytherapy (7/112 patients, 6.3%) had a slightly higher occurrence rate of distant metastasis than surgery (29/775 patients, 3.7%); however, the difference was not statistically significant (p = 0.202). Histologically, 245 cases had cervical lymph node metastasis, which was observed at the initial treatment in 140, while the remaining cases (105 patients) had delayed metastasis. There was a statistically significant difference between the two groups depending on the presence of histological cervical lymph node metastases (*p* < 0.001). Thus, the relationship between cervical lymph node metastases and distant metastases was mainly further investigated. The frequency of distant metastasis was found to be significantly different: 27.3% in patients with 4 or more cervical lymph node metastasis, 50.0% in those with the most distal level (level IV or V) of cervical lymph node metastasis, 26.3% in those with contralateral cervical lymph node metastasis, and 18.0% in those with pathological extranodal extension (ENE). Postoperative chemotherapy and/or irradiation (2.0 Gy/fraction, total 50 Gy) was administered to 155 patients with close or positive margins of primary tumor or ≥ 4 histological cervical lymph node metastases and those positive for ENE.

**Table 2:** Univariate analysis of clinicopathological characteristics and incidence of distant metastasis.

Variables	Incidence of distant metastasis (%)	*p* value
**Sex**		0.081
Male	27/573 (5.1)	
Female	9/354 (2.5)	
**Primary site**		0.003*
PIOC of the mandible	4/16 (25.0)	
Others	32/871 (3.7)	
**Histological differentiation**		< 0.001*
Well/Moderately	26/824 (3.2)	
Poorly	10/63 (15.9)	
**Initial treatment for primary tumor**		0.202
Surgery	29/775 (3.7)	
Brachytherapy	7/112 (6.3)	
**pN**		< 0.001*
No	5/642 (0.8)	
Yes	31/245 (12.7)	
**Number of metastatic nodes**		< 0.001*
0–3	18/821 (2.2)	
≥ 4	18/66 (27.3)	
**Level of metastatic node**		< 0.001*
No metastasis or I + II + III	27/869 (3.1)	
IV + V	9/18 (50.0)	
**Contralateral node metastasis**		< 0.001*
No	27/850 (3.2)	
Yes	10/38 (26.3)	
**ENE**		< 0.001*
No	11/748 (1.5)	
Yes	25/139 (18.0)	
**Postoperative CT and/or RT**		< 0.001*
No	7/732 (1.0)	
Yes	29/155 (18.7)	
Total	36/887 (4.1)	

PIOC, primary intraosseous carcinoma; CT, chemotherapy; RT, radiotherapy; ENE, extranodal extension.

*Significant at *p* < 0.05.

### Site of distant metastasis

Although there was some overlap, the lung was the most common site of distant metastasis (31 patients, 86.1%), followed by the bone (14 patients, 38.9%) and the liver (3 patients, 8.3%) (Table 3). These distant metastases developed between 2 and 94 months after initial treatment (mean, 21.3 months).

**Table 3:** Sites of distant metastasis.

Sites of distant metastasis	Patients**
Lung	31
Bone	14
Liver	3
Mediastinum	2
Others*	3

*Others: Adrenal gland, cerebellum, and spleen, one for each case.

**There are some overlapping cases.

### Identification of high-risk factors for distant metastasis

To identify high-risk factors for distant metastases in all 887 patients, a Cox proportional hazard model was used (Table 4). We identified the following high-risk factors for distant metastasis in patients with OSCC: PIOC of the mandible as the primary site (hazard ratio [HR], 7.200; 95% confidence interval [CI], 2.458–21.091), distal level of neck metastasis being level IV or V (HR, 6.763; 95% CI, 2.934–15.588), and presence of ENE (HR, 8.036; 95% CI, 3.707–17.421).

**Table 4:** Results of the multivariate analysis.

Variables	HR (95% CI)	*p* value
**Primary site**		< 0.001*
PIOC of the mandible	7.200 (2.458–21.091)	
Others	1.000 (Reference)	
**Level of metastatic node**		< 0.001*
No metastasis or I + II + III	1.000 (Reference)	
IV + V	6.763 (2.934–15.588)	
**ENE**		< 0.001*
No	1.000 (Reference)	
Yes	8.036 (3.707–17.421)	

PIOC, primary intraosseous carcinoma; ENE, extranodal extension; HR, hazard ratio; CI, confidence interval.

*Significant at *p* < 0.05.

## Discussion

The rate of distant metastasis of head and neck squamous cell carcinoma (HNSCC) has been reported to be 3.5%–13.7% according to previous reports^2–6^. These studies have revealed high rates of distant metastasis, including cases with uncontrolled primary tumor and/or cervical lymph node metastasis. Interestingly, when only those reports with successful locoregional control of the primary tumor and cervical lymph node metastasis were considered, the rate of distant metastasis was < 10%^5^. In this study, the rate of distant metastasis among patients with locoregional control was 4.1%.

The lung was the most common site of distant metastasis in all previous reports^2–6^, and the findings of this study showed the same result. In general, the bones and liver were the second most common site; however, the site of bone metastases varied, and included the vertebrae, iliac, and humerus. To determine the frequency of distant metastasis, chest computed tomography, followed by a systematic investigation using positron emission tomography, should be performed to confirm diagnosis.

Several studies have attempted to predict distant metastasis by investigating risk factors for distant metastasis, including histological differentiation of the primary tumor^4,5^, tumor stage^3,6^, number of metastatic node^6^, level of cervical lymph node metastasis^2,6^, and ENE^4–6^. In particular, the presence of cervical lymph node metastases has been significantly correlated with the rate of distant metastases^2–6^. In this study, the rate of distant metastasis was significantly higher (12.7%) in patients with cervical lymph node metastasis, and therefore, various aspects of cervical lymph node metastasis were examined in detail. In the multivariate analysis, PIOC of the mandible, level IV or V metastases, and ENE positivity were identified as risk factors for distant metastases. The rates of distant metastases based on the primary site of OSCC have been seldom reported. Previous studies indicated that the rate of distant metastasis in patients with PIOC was as high as 14.7–18.8%^7,8^, which was attributed to extensive tumor infiltration of the mandible already before the initial treatment^9^. To the best of our knowledge, PIOC of the mandible as a risk factor for distant metastasis in OSCC patients may be considered a novel finding. This suggests that considering the high probability of distant metastases when treating PIOC of the mandible, thorough examination using imaging modalities and a more aggressive treatment strategy must be required.

Once distant metastasis is confirmed, prognosis is poor. Therefore, if possible, distant metastases should be prevented or detected at the micro-metastatic stage. The administration of chemotherapy for high-risk groups of distant metastasis is recommended as a preventive measure; however, its effectiveness has not been confirmed. A few reports suggested the possibility of reducing distant metastasis by chemotherapy after locoregional treatment and demonstrated the clinical effect of chemotherapy on tumor cells or micrometastases existing in the blood^10,11^.

Notably, recent studies have reported aggressive treatment for distant metastasis in patients with HNSCC. Daiko et al*.* reported that the 3-year survival rate was 43% after the resection of head and neck carcinoma in patients with lung metastases^12^. The use of cetuximab as a molecular-targeted therapy has been reported to increase the survival rate^1^. Furthermore, the use of immune checkpoint inhibitors, such as nivolumab and pembrolizumab, have resulted in better survival outcomes^13,14^. In this study, patients with postoperative treatment had a higher rate of distant metastasis than those without postoperative treatment. This may have been because postoperative treatment was administered to patients with highly advanced cervical lymph node metastases, such as those with ≥ 4 histological cervical lymph node metastases and those positive for ENE. In patients receiving postoperative radiotherapy, the abscopal effect can be expected to stimulate immune response. This is a rare phenomenon in which radiotherapy causes the tumor to halt its growth even in metastases that are far from the irradiated area. This phenomenon has received renewed attention in head and neck cancer^15,16^. The synergistic impact of the abscopal effect and immune checkpoint inhibitors could aid in activating the immune system and suppressing distant metastases. Further investigation in this regard may allow for new therapeutic findings.

Prevention and early detection of distant metastasis are currently the best options available for OSCC. In the near future, analysis of genetic mutations using liquid biopsy samples may allow us to diagnose distant metastasis early during the initial treatment and ensure timely treatment^17^. In other words, conducting regular systematic checkups in patients with OSCC for risk factors for distant metastasis and implementing appropriate drug therapy should be mandatory.

This study had some limitations. This retrospective study had a small sample size that was insufficient for multivariate analysis for risk factor assessment. It was important to reduce the number of cases excluded from this study by proper management of follow-up data. In addition, improving the local control rate would increase the sample size. We believe that additional follow-up and data collection from a larger population would provide further insights into risk factors for distant metastasis.

In conclusion, this study revealed that in OSCC patients, PIOC of the mandible, cervical lymph node metastasis at levels IV and V, and the presence of ENE were significant risk factors for distant metastasis. In particular, this is the first study to report PIOC of the mandible as a significant risk factor for distant metastasis. When treating patients who are positive for these risk factors, the possibility of occurrence of distant metastases must be considered while planning treatment strategies.

## Methods

This retrospective study included 982 patients with OSCC treated at the Department of Oral and Maxillofacial Surgery in Tokyo Medical and Dental University between January 2008 and December 2017. After excluding patients diagnosed with distant metastasis at initial treatment and metastasis to the oral cavity, those receiving palliative treatment, and those with lack of follow-up data, 941 patients were selected. Among 941 patients with OSCC, those with uncontrolled primary and/or cervical lymph nodes were excluded. Finally, 887 patients with successful locoregional control were analyzed. The duration of observation after initial treatment ranged from 6.5 to 94 months, with an average of 29 months. Statistical analysis of the obtained clinicopathological data was performed in order to identify risk factors for distant metastasis. Fisher’s exact test was used to determine any relevant differences. Multivariate analysis using Cox proportional hazards models was subsequently performed to evaluate variables that were significantly different in the univariate analysis and identify significant risk factors. All statistical analyses were performed using SPSS statistics version 25 for Windows (SPSS Japan Inc., Tokyo, Japan). *p* values < 0.05 were considered statistically significant.

The Institutional Review Board of the Faculty of Dental Hospital of Tokyo Medical and Dental University approved this clinicopathological study and waived the requirement for written informed patient consent owing to the retrospective study design (approval No. D2015-600).

The authors confirm that all experiments were conducted in accordance with the relevant guidelines and regulations.

## Data Availability

All data generated or analyzed during this study are included in this published article.
